# Impact of *apolipoprotein B*-associated cholesterol deficiency genotype on milk composition, somatic cell count, and parity effects in Lithuanian Holstein cows

**DOI:** 10.14202/vetworld.2025.1581-1589

**Published:** 2025-06-16

**Authors:** Ramutė Mišeikienė, Nijolė Pečiulaitienė, Lina Kajokienė, Renata Bižienė, Kristina Morkūnienė, Vilius Marma, Saulius Tušas, Paulius Matusevičius, Ewa Wójcik, Alina Janocha, Anna Milczarek, Laimutis Kučinskas

**Affiliations:** 1 Institute of Biology Systems and Genetic Research, Faculty of Animal Sciences, Lithuanian University of Health Sciences, Kaunas, Lithuania; 2 Institute of Animal Rearing Technologies, Faculty of Animal Sciences, Lithuanian University of Health Sciences, Kaunas, Lithuania; 3 Department of Animal Nutrition, Faculty of Animal Sciences, Lithuanian University of Health Sciences, Kaunas, Lithuania; 4 Institute of Animal Science and Fisheries, Faculty of Agricultural Sciences, University of Siedlce, Siedlce, Poland

**Keywords:** *apolipoprotein B* gene, cholesterol deficiency, genotype, Holstein cows, milk composition, parity, somatic cell count

## Abstract

**Background and Aim:**

Cholesterol deficiency (CD) in Holstein cattle, caused by a loss-of-function mutation in the *apolipoprotein B* (*APOB*) gene, is a heritable autosomal recessive condition with known implications for fat metabolism and cholesterol transport. This study aimed to investigate the effect of the CD genotype on milk yield components, cholesterol concentration, and somatic cell count (SCC) in Lithuanian Holstein cows, and to determine whether lactation number modulates these relationships.

**Materials and Methods:**

A total of 188 cows were classified by lactation: 1^st^ (n = 44), 2^nd^ (n = 50), 3^rd^ and 4^th^ (n = 60), and ≥5^th^ (n = 34). Genotyping for the *APOB* mutation was conducted using allele-specific polymerase chain reaction. Milk fat, protein, lactose, and SCC were determined using LactoScope Fourier-transform infrared spectroscopy and Somascope methods, while cholesterol concentration was measured by high-performance liquid chromatography. Statistical analysis involved the Kruskal–Wallis H test due to non-normal data distribution.

**Results:**

The heterozygous CD genotype was identified in 17.02% of the population, with wild-type and mutant allele frequencies of 0.91 and 0.09, respectively. Non-carriers showed marginally higher fat, protein, and cholesterol levels, with a statistically significant difference in fat content (p = 0.04). When stratified by lactation, significant differences were observed for fat content in the 1^st^ lactation group (p = 0.026), SCC in the 2^nd^ (p = 0.038), and protein content in the 3^rd^ (p = 0.030). No significant variation in milk cholesterol concentration was detected across genotype groups in any lactation group.

**Conclusion:**

This study confirms the presence of the CD-associated *APOB* allele in the Lithuanian Holstein population. While CD status significantly influenced milk fat percentage, its effect on other milk composition traits and SCC was limited. Parity exhibited specific but non-consistent modulating effects. Further large-scale, longitudinal studies are warranted to elucidate the physiological underpinnings of these findings.

## INTRODUCTION

Genetic disorders represent a significant challenge in the dairy industry, primarily due to an increase in inbreeding resulting from intensive selection strategies and the widespread use of elite sires [1]. Over 50 genetic defects and traits have been identified within the Holstein cattle breed [2]. One of the most recently discovered genetic anomalies is a haplotype associated with cholesterol deficiency (HCD) [3, 4]. This disorder is caused by a 1233 bp insertion in the fifth exon of the *apolipoprotein B* (*APOB*) gene on *Bos taurus* autosome 11, leading to a frameshift mutation and premature protein truncation [3]. The *APOB* gene encodes *APOB*, the primary apolipoprotein in chylomicrons and low-density lipoproteins [3]. While heterozygous carriers of this mutation are phenotypically normal, they exhibit reduced plasma cholesterol and lipoprotein levels [5]. This loss-of-function mutation in *APOB* is inherited in an autosomal recessive manner and causes cholesterol deficiency (CD), which adversely affects calf survival and development [6, 7]. Given its role in cholesterol metabolism, mutations in *APOB* can influence cholesterol levels across various species, including cattle.

Globally, the prevalence of HCD genetic defects is considerable, ranging between 6% and 17% [8]. In Germany, approximately 9% of the Holstein population are heterozygous carriers of the *APOB* mutation [9, 10]. Schütz *et al*. [4] estimated carrier frequencies at up to 5% in both European and North American Holstein populations. In Canada, the estimated prevalence reaches approximately 16% [11], while Zhang *et al*. [12] identified a 3.62% carrier rate among 1633 Holstein cows in China. However, the frequency of the CD genotype in Lithuania’s Holstein population remains unknown due to limited prior studies.

Milk cholesterol content varies among species and breeds and is influenced by genetic background, nutritional factors, lactation stage, and breed characteristics [13–16]. In cows, both genetic and environmental factors play a role in determining milk cholesterol levels [17]. Notably, HCD carriers often exhibit higher genetic merit for milk protein, fat, and somatic cell scores than non-carriers [7, 18]. Genetic components contribute to 10%–18% of the observed phenotypic variability in milk cholesterol concentration [19]. Milk cholesterol is also influenced by lactation stage, seasonality, somatic cell count (SCC), and fat content, with cholesterol levels positively correlated with SCCs [17].

The physiological impact of the *APOB*-associated CD mutation on dairy cows, particularly heterozygous carriers remains inadequately studied [6]. Some findings suggest that milk yield and its compositional traits are not significantly affected by carrier status [5]. Given that the Holstein breed originated from a limited genetic base, regional populations exhibit varying genetic loads, reflecting differing local selection practices [20]. The effectiveness of genetic improvement depends on the precision of selection, which is contingent on the accuracy of breeding value estimations and the differentiation among genetically diverse animals. Genetic gain can be optimized not only by increasing phenotypic data collection but also by incorporating genomic information. The prediction of genomic breeding values allows for more accurate selection, thereby enhancing the efficiency of genetic improvement programs [21, 22].

Despite the growing body of literature on the *APOB*-associated CD mutation in Holstein cattle, significant knowledge gaps persist regarding its phenotypic effects in heterozygous carriers, particularly within underrepresented regional populations such as Lithuania. Most existing studies have primarily focused on homozygous-affected calves, emphasizing severe metabolic impairments and mortality outcomes. However, the physiological and productive implications of heterozygous carriage, especially regarding milk composition traits and SCC, remain inadequately explored. Furthermore, prior investigations have reported inconsistent findings on the impact of CD carrier status on fat, protein, and cholesterol levels in milk, with some studies indicating elevated genetic merit in carriers while others reveal negligible or adverse effects. There is also limited understanding of how parity (lactation number) interacts with CD genotype to influence milk yield components and udder health indicators across different lactations. In Lithuania, no prior study has systematically evaluated the prevalence of the *APOB* CD allele or its association with milk production traits under herd management and feeding practices. Thus, comprehensive regional data are lacking to inform genetic selection programs, dairy health management, and breeding strategies targeting metabolic efficiency and milk quality.

This study was designed to assess the impact of the heterozygous *APOB* genotype associated with CD on key milk production traits, including fat, protein, lactose content, cholesterol concentration, and SCC in Lithuanian Holstein cows. In addition, the study aimed to investigate whether the effect of CD carrier status varies across different lactation numbers, thereby elucidating potential genotype-parity interactions. By quantifying allele frequency and evaluating the phenotypic consequences of carrier status, this research provides foundational insights into the metabolic and productive implications of *APOB*-associated CD under field conditions. The findings aim to support the refinement of breeding strategies and contribute to more informed genomic selection practices in dairy herds, particularly in Eastern European contexts where such data remain scarce.

## MATERIALS AND METHODS

### Ethical approval

The study was conducted in accordance with the methodology outlined in the Law on the Welfare of Farm Animals of the Republic of Lithuania and complied with Directive 2010/63/EU of the European Parliament and of the Council on the protection of animals used for scientific purposes. Ethical approval was obtained for the study (Ethics approval No. 2024-BEC2-1055).

### Study period and location

The study was conducted at a dairy farm located in the Kaunas district from October 2023 to April 2024. Milk analysis was performed at the Central Milk Testing Laboratory of the Joint Stock Company *Pieno Tyrimai* (Kaunas, Lithuania). The determination of milk cholesterol content was carried out at the Institute of Animal Rearing Technologies, Lithuanian University of Health Sciences. Molecular genetic analysis was conducted at the Dr. K. Janušauskas Laboratory of Animal Genetics, also part of the Lithuanian University of Health Sciences.

### Animals and cross-section study design

A total of 188 cows were included in this cross-sectional study: 44 cows in the first lactation (1^st^ lactation group), 50 cows in the second lactation (2^nd^ lactation group), 18 and 42 cows in the third and fourth lactations, respectively (3^rd^ lactation group), and 34 cows in the fifth and older (4^th^ lactation group). The cows were in the early stage of lactation (up to 100 days). On the research farm, the average cow produces 9591 kg of milk, with a fat content of 4.02% and a protein content of 3.43%. The animals were housed on rubber mats throughout the year. The cows were fed a nutritionally balanced total mixed ration comprising grass silage, maize silage, hay, and concentrate, formulated to meet the physiological demands of a 600 kg cow producing approximately 25 kg of milk per day. All animals were maintained in compliance with established hygiene standards. Milking was carried out twice daily using a side-by-side milking parlor.

### DNA extraction and polymerase chain reaction (PCR)

A total of 188 Lithuanian Holstein cows were tested for the *APOB* gene mutation. Hair follicle samples were collected from each animal. Genomic DNA was extracted from these hair follicles using the Chelex DNA extraction method, which involved 200 μL Chelex-100, 7.5 μL DTT (1 M), and 10.7 μL Proteinase K (20 mg/mL) [23]. Following DNA extraction, proteinase K was inactivated at 94°C for 10 min. The DNA concentration of samples ranged from 60 ng/μL to 140 ng/μL. Genotyping was conducted according to the protocol described by Kamiński and Ruść [24].

PCR was performed in a 27 μL reaction mixture containing 12.5 μL DreamTaq PCR Master Mix (Thermo Fisher Scientific Baltics, Vilnius, Lithuania), two forward primers (18.1 nM and 18.9 nM) and one reverse primer (13.6 nM), 1 μL of each primer (Thermo Fisher Scientific Baltics), 2.5 μL of nuclease-free water, and 10 μL of genomic DNA. Amplification was performed using the GeneAmp PCR System 2700 (Applied Biosystems, Foster City, CA, USA). To identify carriers of the recessive CD mutation in the *APOB* gene (NC_037338.1), animals were selected following criteria established in previous studies by Kamiński and Ruść [24] and Nicholas *et al*. [25].

Animals were genotyped using allele-specific primers: two forward primers and one reverse primer. The first forward primer amplified a 249 bp fragment representing the wild-type allele, while the second amplified a 436 bp fragment indicative of the long terminal repeat insertion in the *APOB* gene. The PCR product sizes and primer sequences are provided in Table 1. PCR products were separated by electrophoresis on a 2% agarose gel in 1× Tris-acetate-EDTA buffer, stained with 10 μL ethidium bromide (CS-300V Cleaver Scientific Ltd., London, UK), and electrophoresed at 100 V for 50 min. Fragment visualization and sizing were conducted under ultraviolet (UV) light using the MiniBisPro documentation system (DNA Bio-Imaging System, Neve Yamin, Israel).

**Table 1:** Primers, sequence of PCR reaction conditions, size of PCR product, and genotype patterns.

Primer sequence (5′ - 3′)		PCR profile (35 cycles)		PCR product size
Forward (Wild) Forward (Mutant) Reverse	GGTGACCATCCTCTCTCTGC CACCTTCCGCTATTCGAGAG AGTGGAACCCAGCTCCATTA	94°C	4 min	Normal: 249 bp Carrier: 249, 436 bp
		94°C	1 min	
		62°C	30 s	
		72°C	1 min	
		72°C	4 min	

PCR=Polymerase chain reaction

### Milk composition, SCC, and cholesterol analysis

Composite milk samples were collected from all four quarters of each of the 188 cows during the evening milking session. Each sample was divided into two equal parts: one for the assessment of milk production and quality indicators (fat, protein, lactose, and SCC) and the other for cholesterol analysis. All samples were collected in sterile tubes during control milking and immediately transported to the laboratory for analysis.

Genetic parameters for milk quality (SCC, measured in thousands/mL) and composition (fat, protein, and lactose percentages) were evaluated for the Lithuanian Holstein population. Analyses were conducted at the Central Milk Testing Laboratory of *Pieno Tyrimai* (Kaunas, Lithuania), in accordance with the LST ISO 17025 standard. Data for SCC and milk composition traits were derived from monthly test milking records. Fat, protein, and lactose contents were quantified using the LactoScope Fourier-transform infrared spectroscopy instrument (Perten Instruments, Stockholm, Sweden). SCC was determined using the Somascope analyzer (Perten Instruments), employing the fluoro-optoelectronic method.

For cholesterol analysis, composite milk samples were processed following a modified method of Polak *et al*. [26]. Each 1.00 ± 0.01 g milk sample was mixed with 5 mL of saturated potassium hydroxide and incubated at 60°C for 60 min. After cooling, the analyte was extracted twice with 5 mL of hexane. To break the emulsion, 3 mL of distilled water was added. The samples were then centrifuged at 1,000 × g for 2 min (Sigma 2–5, Germany). The combined extract was dried under a nitrogen stream and reconstituted in 1 mL of the mobile phase. Cholesterol quantification was performed using a Varian ProStar high-performance liquid chromatography system (Varian Corp., Palo Alto, CA, USA), comprising two ProStar 210 pumps, a ProStar 410 autosampler, a ProStar 325 UV visible detector, and Galaxy software (Agilent, Santa Clara, CA, USA). A Discovery^®^ HS C18 column (150 × 4.6 mm, 5 μm; SupelcoTM Analytical, Bellefonte, PA, USA) was used. The mobile phase consisted of 2-propanol and acetonitrile in a 45:55 ratio, with a flow rate of 1.0 mL/min. Detection was performed at 210 nm. Results were expressed in milligrams per gram of sample.

### Statistical analysis

The Shapiro–Wilk test was used to assess the normality of data distribution. Since the data did not follow a Gaussian distribution, the Kruskal–Wallis H test was employed to achieve the study objectives. This test was applied to evaluate differences in individual genotype traits (such as fat and protein content) across various lactation groups. All statistical analyses were performed using IBM Statistical Package for the Social Sciences Statistics for Macintosh, Version 28.0 (IBM Corp., NY, USA).

## RESULTS

### Genotyping results for APOB mutation

**Figure 1:**
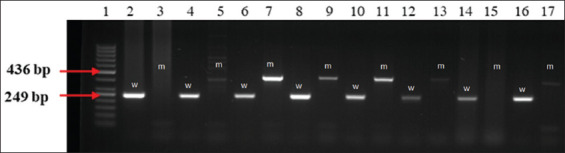
Identification of heterozygous cholesterol deficiency genotypes in cows. Lane 1–50 bp DNA ladder marker (Thermo Fisher Scientific Baltics). Lanes 6–7, 8–9 and 10–11 are heterozygous genotypes (two fragments were amplified–436 bp and 249 bp). Lanes 2–3, 4–5, 12–13, 14–15, 16–17 – normal animals (one fragment of 249 bp was amplified); (w=Wild, m=Mutant). For the analysis of a single DNA sample, two wells were used: one with forward primer F1 (wild) and reverse primer R1 and another with forward primer F2 (mutant) and reverse primer R. Polymerase chain reaction amplification was performed in these two separate wells, resulting in product lengths of 249 bp for the wild type and 436 bp for the mutant.

The results of PCR genotyping for the *APOB* gene mutation are illustrated in Figure 1. In wild-type animals, a single 249 bp fragment was amplified, whereas heterozygous carriers of the CD mutation exhibited two distinct fragments of 436 bp and 249 bp. Among the 188 cows analyzed, 32 were identified as heterozygous carriers, accounting for 17.02% of the total population. The calculated allele frequencies were 0.91 for the wild-type allele and 0.09 for the mutant allele in the studied population. Genotyping procedures included both internal positive and negative controls. In addition, at least 10% of the samples were replicated to assess the reproducibility of the genotyping process, which exceeded 99%.

### Descriptive analysis of milk parameters by CD genotype

All 188 milk samples analyzed in the study were classified into two groups based on CD status: 156 non-carriers and 32 carriers. Descriptive statistics for milk production traits, SCC, and cholesterol content by CD status are presented in Table 2. Non-carrier cows exhibited marginally higher levels of milk cholesterol, protein, and fat compared to heterozygous carriers. Specifically, the median fat percentage in milk from heterozygous cows was 0.34% lower than that of non-carrier cows with the same mutation (p = 0.04). No statistically significant differences were observed for the remaining parameters between carriers and non-carriers.

**Table 2:** Summary statistics for milk indicators, SCC, and cholesterol content in milk, and relation of cows’ CD status with analysed indicators.

Parameter	Group	Median	Minimum	Maximum	df	F	p-value
Fat (%)	Total average	4.3300	3.07	6.69	1	4.233	0.040*
Non-carrier	4.3900	3.07	6.69			
	Carrier	4.0500	3.20	5.29			
Protein (%)	Total average	3.5000	3.08	4.61	1	1.507	0.220
	Non-carrier	3.5300	3.08	4.36			
	Carrier	3.4650	3.11	4.61			
Lactose (%)	Total average	4.4900	3.66	4.91	1	0.001	0.980
	Non-carrier	4.5000	3.66	4.91			
	Carrier	4.4350	4.26	4.70			
Somatic cell count (thousand/mL)	Total average	181.00	11.00	1411.00	1	0.224	0.636
	Non-carrier	180.00	11.00	1411.00			
	Carrier	230.00	18.00	662.00			
Cholesterol content in milk (mg/100 g)	Total average	9.4000	6.80	13.90	1	0.047	0.829
	Non-carrier	9.3000	6.80	13.90			
	Carrier	9.5500	7.60	11.80			

* p < 0.05, df=Degree of freedom, SCC=Somatic cell count, CD=Cholesterol deficiency

### Effect of parity on milk traits in CD carriers and non-carriers

**Figure 2:**
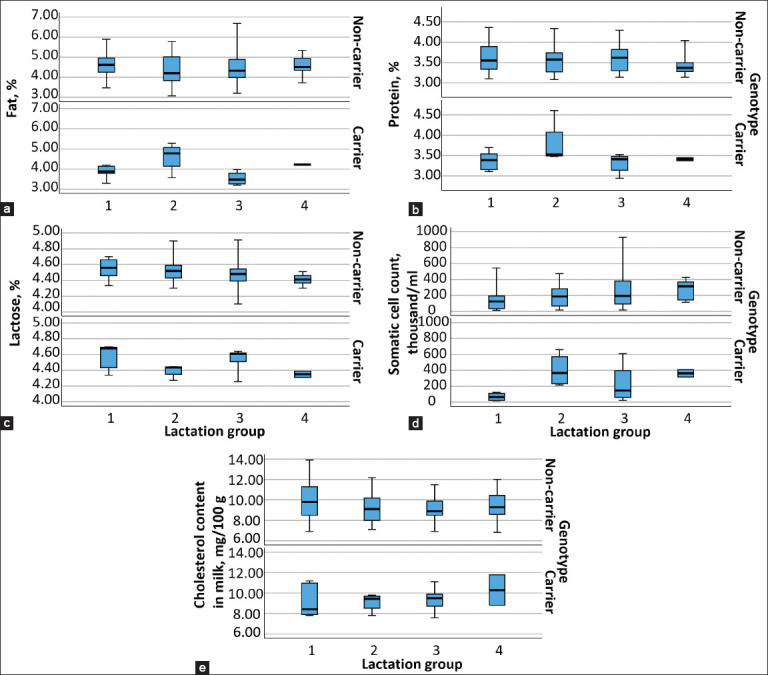
(a-e) Boxplots for milk production, somatic cell count, and cholesterol content in milk data, according to the lactation groups by CD status of cows (the study included 188 cows: 44 cows in the 1^st^ lactation group (34 non-carriers, 10 carriers); 50 cows in the 2^nd^ lactation group (42 non-carriers, 8 carriers); 60 cows in the 3^rd^ lactation group (50 non-carriers, 10 carriers) and 34 cows in the 4^th^ lactation group (30 non-carriers, 4 carriers).

The study further aimed to assess whether lactation number influenced milk productivity traits, SCC, and cholesterol content when considering CD genotype status. Descriptive statistics stratified by lactation number and CD status are illustrated in Figure 2. Boxplot analyses indicated that in lactation groups 1, 3, and 4, carrier cows generally produced milk with lower fat and protein percentages. In contrast, carriers in the first lactation group exhibited a slightly higher lactose percentage compared to non-carriers. Carrier cows in lactation groups 2 and 4 had elevated SCC values compared to their non-carrier counterparts. Among non-carrier cows, the mean SCC tended to increase progressively with lactation number. The lowest cholesterol concentration was recorded in milk from heterozygous cows in the first lactation group.

### Statistical differences in milk composition across lactation groups

As shown in Table 3, differences in milk fat, protein, lactose, SCC, and cholesterol concentrations between carriers and non-carriers across lactation groups were largely non-significant (p > 0.05), with several exceptions. Statistically significant differences were identified in fat content within the first lactation group (p = 0.026), SCC in the second lactation group (p = 0.038), and protein content in the third lactation group (p = 0.030). Furthermore, CD status did not have a significant effect on milk cholesterol concentration in any of the lactation groups.

**Table 3:** Influence of cholesterol deficiency status of cows on milk composition parameters, somatic cell count, and cholesterol content in different lactation groups.

Parameter	F	p-value
1 lactation group		
Fat (%)	4.988	0.026
Protein (%)	1.291	0.256
Lactose (%)	0.616	0.432
Somatic cell count (thousand/mL)	0.043	0.836
Cholesterol content in milk (mg/100 g)	0.554	0.457
2 lactation group		
Fat (%)	0.198	0.656
Protein (%)	0.445	0.505
Lactose (%)	3.182	0.074
Somatic cell count (thousand/mL)	4.309	0.038
Cholesterol content in milk (mg/100 g)	0.006	0.941
3 lactation group		
Fat (%)	2.606	0.106
Protein (%)	4.714	0.030
Lactose (%)	1.571	0.210
Somatic cell count (thousand/mL)	0.375	0.540
Cholesterol content in milk (mg/100 g)	0.012	0.911
4 lactation group		
Fat (%)	1.800	0.180
Protein (%)	0.139	0.709
Lactose (%)	1.101	0.294
Somatic cell count (thousand/mL)	0.356	0.551
Cholesterol content in milk (mg/100 g)	0.356	0.551

p – significance of F statistics

## DISCUSSION

### Prevalence of HCD and *APOB* allele frequency

Analysis by various authors has demonstrated a significant prevalence of HCD in Holstein cattle worldwide. Studies reported HCD carrier frequencies ranging from approximately 5%–9% in Europe and North America [4, 9, 24], and up to 16% in Canada [11]. In Lithuania, prior data on the prevalence of this genetic defect in the Holstein population have been lacking. This study confirmed the presence of the recessive allele associated with CD in the Lithuanian Holstein population, with an observed allele frequency of 0.09, suggesting a potential for nationwide distribution.

### Effect of CD status on milk composition and SCC

In contrast to the findings of Wang *et al*. [7] and Cole *et al*. [18], our results revealed that heterozygous carriers produced milk with lower fat and protein percentages. This observation, however, is partially consistent with Cole *et al*. [18], who also reported phenotypic alterations associated with CD status. In line with Wang *et al*. [7], our findings also indicated higher SCCs in heterozygous carriers compared to non-carriers, with an average increase of 50,000/mL.

### Cholesterol content in milk and relationship to CD genotype

Changes in blood cholesterol levels are known to influence its concentration in milk [17]. Piironen *et al*. [27] reported cholesterol concentrations ranging from 5.6 mg to 6.4 mg/100 g in semi-fat (1.5%) cow milk and up to 11.2 mg/100 g in full-fat (3%) milk. The median milk cholesterol content in our studied herd was 9.4 mg/100 g. Our results suggest that CD status did not significantly affect milk cholesterol content across lactation groups, with the exception of milk fat percentage. This aligns with the findings of Wang *et al*. [7], indicating that heterozygous CD carriers generally do not exhibit statistically significant differences in milk production traits, apart from isolated exceptions.

### Influence of lactation number on milk traits in CD carriers

Strzałkowska *et al*. [14] have noted that milk cholesterol levels are influenced by the stage of lactation, whereas parity (lactation number) may not be a significant factor. In our study, however, significant differences were observed between CD carriers and non-carriers in specific lactation groups: milk fat content in the first lactation group, SCC in the second, and milk protein content in the third (p < 0.05). These findings suggest that parity may interact with CD status in a lactation-stage-specific manner, though this requires further confirmation in larger cohorts. Strzałkowska *et al*. [17] similarly found no positive association between lactation number and cholesterol levels in cow milk.

### Potential mechanisms of *APOB* mutation effects

The *APOB*-associated CD haplotype may not exert a direct phenotypic effect on milk yield or composition, but rather influence underlying metabolic pathways. Gross *et al*. [5] used specific abbreviations to define genetic groups and found significant differences in cholesterol and lipoprotein concentrations between heterozygous carriers (CDC, genetic group with one mutated allele and one normal allele) and wild-type animals (CDF, animals with two normal alleles – the “wild-type” genotype), yet reported no differences in other metabolic indicators, milk yield, or reproductive performance. In addition, Gross *et al*. [6] raised the question of whether the CD mutation impairs cholesterol biosynthesis and metabolism, or whether its physiological impact stems solely from disrupted lipid absorption and transport due to a lack of *APOB* necessary for lipoprotein assembly.

No significant differences in clinical mastitis incidence were observed between heterozygous and homozygous animals [6]. Conversely, Cole *et al*. [18] reported a minor favorable genetic association between the CD-associated haplotype and somatic cell score, a proxy indicator for mastitis susceptibility. The *APOB* haplotypes may modulate immune or inflammatory responses through altered lipid metabolism, thus influencing SCC [28].

### Complex interactions affecting milk traits and health parameters

Genetic variations in the *APOB* gene can affect cholesterol metabolism and have downstream effects on milk composition and SCC. The observed relationships between *APOB* haplotypes, milk traits, SCC, and cholesterol levels appear to be multifactorial and modulated by interactions with environmental conditions, management practices, and other genomic loci. These findings highlight the complexity of interpreting phenotypic effects arising from single-gene mutations in the broader context of dairy cow physiology and productivity.

## CONCLUSION

This study represents the first report on the prevalence and phenotypic impact of the *APOB*-associated CD allele in the Lithuanian Holstein dairy cow population. Genotyping of 188 cows revealed a heterozygous carrier frequency of 17.02%, with an estimated allele frequency of 0.09, confirming the presence of this recessive mutation within the national herd.

Heterozygous carriers exhibited significantly lower milk fat content compared to non-carriers (p = 0.04), and descriptive trends also suggested marginal reductions in protein and cholesterol concentrations. When stratified by lactation stage, statistically significant differences were observed in specific lactation groups: fat percentage in the 1^st^ (p = 0.026), SCC in the 2^nd^ (p = 0.038), and protein content in the 3^rd^ (p = 0.030). However, the CD status did not significantly influence milk cholesterol levels across lactation groups, and overall effects on milk composition were limited, corroborating previous findings that CD carriers often remain phenotypically indistinct except for isolated traits.

Practical implications of this research suggest that while the CD allele may not severely impair productivity in heterozygous carriers, its presence in the breeding population warrants monitoring, especially in the context of animal health, milk quality, and SCC, which is a key udder health indicator. Integrating genotypic screening into breeding programs could aid in mitigating the propagation of the CD allele, especially in high-performing Holstein herds.

Strengths of this study include its comprehensive design, combining genotyping, detailed milk composition analysis, and parity-stratified assessment in a controlled farm environment. The study also applied validated laboratory and statistical methods to ensure data reliability.

Limitations include the relatively modest sample size, single-farm setting, and the absence of homozygous individuals, which restricts generalizability and the ability to assess the full phenotypic spectrum of CD.

Future research should prioritize large-scale, multi-herd studies involving broader genetic backgrounds, longitudinal monitoring across complete lactation cycles, and integration of other metabolic and immune parameters. Functional studies exploring gene environment interactions and molecular mech-anisms underlying lipid transport defects are also recommended.

In summary, while the *APOB* CD allele is present in the Lithuanian Holstein population at a moderate frequency, its effect on most milk production traits appears limited. Nonetheless, targeted genomic surveillance may support more informed breeding decisions, contributing to improved herd genetic health and milk quality assurance.

## DATA AVAILABILITY

All the generated data are included in the manuscript.

## AUTHORS’ CONTRIBUTIONS

RM: Designed the study and drafted the manuscript. NP, KM, LK, PM, and RB: Analyzed the results and revised the manuscript. RM, ST, and PM: Conducted the study and data collection and analysis. VM: Conducted the statistical analysis. EW, AJ, and AM: Conducted a literature search and data analysis. LK: Reviewed and revised the manuscript. All authors have read and approved the final manuscript.
